# Long-term outcomes of transcatheter Potts shunt in children with suprasystemic pulmonary arterial hypertension

**DOI:** 10.3389/fcvm.2022.1028304

**Published:** 2022-10-26

**Authors:** Raymond N. Haddad, Maryline Levy, Isabelle Szezepanski, Sophie Malekzadeh-Milani, Damien Bonnet

**Affiliations:** ^1^Centre de Référence Malformations Cardiaques Congénitales Complexes—M3C, Hôpital Universitaire Necker-Enfants Malades, AP-HP, Paris, France; ^2^Université de Paris Cité, Paris, France

**Keywords:** children, heart failure, outcomes, Potts shunt, pulmonary arterial hypertension

## Abstract

**Background:**

Transcatheter Potts shunt (TPS) is a palliation alternative for children with severe pulmonary arterial hypertension (PAH). Debates on the long-term outcomes remain unsolved.

**Objectives:**

To evaluate long-term clinical and procedural outcomes of TPS intervention.

**Methods:**

Single-center retrospective data review of children with severe PAH who had TPS between 2009 and 2018. Patients who died per-operatively and early post-procedure were excluded. Long-term outcomes of survivors were evaluated.

**Results:**

Out of 13 identified patients (53.8% males), 7 had endovascular stenting of probe/patent arterial ducts, while 6 individuals had aorta-to-pulmonary radiofrequency perforation and covered stent placement. Compared to baseline, the overall clinical condition significantly improved at discharge (*p* < 0.001) and stayed better at the last visit (*p* < 0.05) despite frequent clinical worsening events across follow-up. Improvement in functional class across follow-up was significant (*p* < 0.001). There was, however, no significant improvement in other disease markers (TPASE, 6MWD z-scores, and NT-proBNP levels) or reduction in PAH medications. The median follow-up was 77.4 months (IQR, 70.7–113.4). Survival was 100% at 1 year and 92.3% at 6 years. Freedom from reinterventions was 77% at 1 year and 21% at 6 years. Nine (69.2%) patients had stent reinterventions at a median of 25 months (IQR, 9.5–56) postoperative. Balloon dilatation and restenting were performed in 53.8% and 46.2% of patients, respectively. High-pressure post-dilatation of implanted stents was performed in 53.8% of patients during TPS intervention for incomplete stent expansion and/or residual pressure gradient and was associated with higher rates of reinterventions (*p* = 0.021). Stent malfunctioning was present in 46.2% of patients at last follow-up. Two patients are listed for heart-lung transplantation.

**Conclusion:**

Survivors of TPS procedures experience significant improvement in functional class that can be durable. Clinical worsening and stent malfunctioning are frequent morbid events indicating recurrent transcatheter reinterventions throughout follow-up. Six-year survival is, however, satisfactory.

## Introduction

Pulmonary arterial hypertension (PAH) is a chronic disease and the prognosis remains guarded despite profound advances in medical therapies (1, 2). The revival of the surgical Potts shunt as palliation of PAH in children was an interesting innovation coming from collaborative research in Paris hospitals (3–5). The outcomes of this surgical procedure were promising inspiring interventionists to duplicate the technique using transcatheter technologies (6, 7). Transcatheter Potts shunt (TPS) was initially performed by stenting a restrictive arterial duct with effective results (6, 8). However, in the absence of an arterial duct, the *de novo* percutaneous shunt creation between the left pulmonary artery (LPA) and descending aorta (DAO) was technically challenging and not complication-free (7, 9). The technical aspects and early outcomes of both procedures were described in several studies (6–9). Early morbidity and mortality benefits were reported but these conclusions were mainly derived from studies of mixed populations with surgical and transcatheter shunts, keeping debates regarding TPS ongoing (2, 10, 11). The international Potts shunt registry recently underscored that TPS was a risk factor for early mortality (12). The data of this registry also showed that the type of shunt, and in particular transcatheter shunts, did not prove a significant risk factor for late events. The technical issues of TPS, in the long run, were not addressed in the registry study. Therefore, we expand upon these earlier findings to evaluate the long-term outcomes of a large single-center experience with TPS and focus on the durability of the implanted stents in procedure survivors.

## Patients and methods

### Study design

We retrospectively reviewed and included all children with suprasystemic PAH who survived ductal and non-ductal TPS procedures at our institution from October 2009 to June 2018. All patients were assigned to group 1 according to the classification of World Symposium on Pulmonary Hypertension (WSPH). Four patients had non-ductal TPS procedures and died either per-operatively, in-hospital, or within 4 weeks after hospital discharge. These patients have been reported in previous cohorts and were excluded from this study (7, 9, 13). They had pre-operative World Health Organization functional class (WHO-FC) IV with severe right ventricular systolic dysfunction requiring elective or rescue veno-arterial ECMO support.

All children had detailed investigations and received supportive therapy including diuretics, oral anticoagulation, and oxygen on a case-by-case basis. Pre-operative demographics, invasive hemodynamics, and procedural data were collected from the medical records, and catheterization reports. We specifically collected WHO-FC, presence of syncope, upper and lower limb oxygen saturations, 6-min walk distance (6MWD) values and z-scores (14), N-terminal pro-brain natriuretic peptide (NT-proBNP) levels, Tricuspid Annular Plane Systolic Excursion (TAPSE) values and z-scores (15), maximal velocity on implanted stents, number, dose, and type of PAH specific medical therapies at baseline, hospital discharge, 6 and 12 months follow-up and last available follow-up to be evaluated and compared. Specific PAH therapies including oral endothelin receptor antagonists (ERA), phosphodiesterase type-5 inhibitors (PDE5I), and subcutaneous or intravenous prostanoid therapy were used either as sequential add-on therapy or as up-front triple therapy according to clinical status at baseline and worsening events during the follow-up.

### Procedure

All patients underwent repetitive pre-operative detailed clinical and echocardiographic evaluations. Candidates for TPS were children with supra-systemic PAH, non-responder during acute vasoreactivity testing, along with worsening WHO-FC, and/or insufficient improvement in clinical signs and symptoms despite maximal doses of at least a combination of PDE5I and ERA for at least 6 months before the TPS procedure. Patients with one PAH therapy and immediately sent for TPS had restrictive arterial ducts and presented special socioeconomic conditions (i.e., living abroad, family decision) motivating the earlier indication for TPS. The Potts shunts created in the catheterization laboratory included: (1) endovascular stenting of a restrictive or probe arterial duct (i.e., ductal TPS); and (2) *de novo* shunt generation between the LPA and the DAO with a direct radiofrequency puncture and subsequent placement of a balloon-expandable covered stent (i.e., non-ductal TPS). Both TPS procedures were previously detailed (6, 7). In case of incomplete stent expansion and/or residual pressure gradient > 10 mmHg across the stent, stent re-dilation was performed using appropriately sized angioplasty high-pressure balloons to reduce or abolish the pressure gradient. Patients who had ductal stenting were prescribed single antiplatelet therapy while those who had non-ductal TPS were prescribed dual antiplatelet therapy with aspirin and clopidogrel. Patients were transferred to the ICU for close monitoring when indicated.

### Follow-up protocol

Routine outpatient follow-ups were scheduled for 1, 3, 6, and 12 months post-procedure and yearly thereafter. Assessment included clinical evaluation of clinical status, physical examination (with a focus on clinical signs of right ventricular failure), saturation measurements, transthoracic echocardiography, lab tests, and 6MWD test. Improvement in WHO-FC, shunting across the Potts shunt, quantitative improvement in right ventricular function (i.e., increase in TAPSE scores), and decrease in NT-proBNP levels were used as criteria to modify the PAH medical therapies during follow-up. We defined clinical worsening by the presence of at least one of the following criteria: (1) worsening WHO-FC; (2) rising NT-pro BNP levels ≥ 1,400 pg/mL; (3) decreasing TAPSE score ≤ 15 mm; (4) dose increase or adjunction of diuretics; and (5) adjunction of new PAH-specific medical therapy. Patients were classified in a good overall clinical condition in the absence of any aspect of clinical worsening or lack of improvement. We defined stent malfunctioning as Doppler maximum velocity on the implanted stent > 2 m/s. Follow-up cardiac catheterization (hemodynamic and/or interventional) was performed in case of documented stent malfunctioning and/or clinical worsening. Percutaneous interventions on the stent included balloon dilation and endovascular re-stenting and were performed in case of a significant invasive pressure gradient (i.e., ≥ 10 mmHg) (7). Stent fracture and degree of neo-intimal proliferation were also looked at.

### Statistical analyses

Statistical analyses were performed using SPSS, Version 22.0 for Macintosh (IBM, Armonk, NY, USA). Categorical variables were reported as frequency and percentage and continuous variables were represented as median with interquartile range. Statistical analysis for categorical variables was conducted using Fisher’s exact test. Distribution of continuous variables was compared using Mann Whitney *U*-test. A *p*-value < 0.05 was considered statistically significant. All reported *p*-values are two-sided. Kaplan-Meier univariate analyses were generated to show event-free rates.

## Results

### Baseline patients characteristics

We identified 13 children (53.8% males) discharged alive after TPS. Patient characteristics are shown in Table 1. The median age and weight at the time of the intervention were 8.7 years (IQR, 5.7–12) and 26.8 kg (IQR, 18–34.7), respectively. The median time from PAH diagnosis to TPS was 2.5 years (IQR, 1.4–5.8). All children had asthenia and growth retardation. At the time of the procedure, 54% of patients were receiving triple therapy, 23% dual therapy, and 23% monotherapy. More specifically, 92.3% of children were receiving a PDE5I, 84.6% were receiving an ERA, and 53.8% were receiving either intravenous or subcutaneous infusions of prostanoids. When referred for the TPS procedure, 69.2% of the children were considered WHO-FC III or IV. Pre-operative right heart catheterization documented supra-systemic right ventricular pressures in all patients (Table 1).

**TABLE 1:** Baseline clinical and procedural characteristics.

	Total, *n* = 13	Group 1, *n* = 6	Group 2, *n* = 7	*P-value*
Male, *N* (%)	7 (53.8)	3 (50)	4 (57.1)	1^a^
**Demographics at diagnosis**				
Age (years), *median (IQR)*	3.67 (0.79, 9.75)	6.29 (2.08, 11.44)	2.58 (0.67, 5.75)	0.445^b^
Weight (Kg), *median (IQR)*	14.7 (6.9, 22.5)	18.5 (10.6, 34.25)	13 (6.7, 21)	0.534^b^
Oxygen saturation (%), *median (IQR)*	96 (91.5, 97.5)	96.5 (95.5, 98.5)	93 (89, 97)	0.138^b^
Cyanosis, *N* (%)	3 (23.1)	–	3 (42.9)	0.192^b^
Neurological comorbidity, *N* (%)	4 (30.8)	3 (50)	1 (14.3)	0.07^a^
Associated hemodynamically	4 (30.8)	–	4 (57.1)	0.192^a^
significant CHD, *N* (%)				
Idiopathic pulmonary arterial hypertension, *N* (%)	4 (30.8)	2 (33.3)	3 (28.6)	1^a^
Heritable pulmonary arterial hypertension, *N* (%)	7 (53.8)	4 (66.7)	3 (42.9)	0.592^a^
BMPR2 mutation, *N* (%)	4 (30.8)	4 (66.7)	–	**0.021^a^**
TBX4 mutation, *N* (%)	1 (7.7)	–	1 (14.3)	–
Down syndrome, *N* (%)	2 (15.4)	–	2 (28.6)	0.462^a^
Delay from diagnosis to intervention (years), *median (IQR)*	2.49 (1.45, 5.83)	5.06 (2.41, 6.71)	1.88 (0.7, 4.99)	0.138^b^
**Demographics at intervention, *median (IQR)***				
Age (years)	8.67 (5.66, 12)	9.83 (8.67, 14.39)	8.08 (2.25, 9.67)	0.101^b^
Weight (kg)	26.8 (18, 34.7)	34.5 (26.35, 47.1)	22 (12.5, 31)	**0.022^b^**
Height (cm)	133 (108, 140.5)	134.5 (130, 148)	122 (88, 140)	0.181^b^
BSA (m^2^)	0.97 (0.72, 1.17)	1.16 (0.96, 1.38)	0.84 (0.55, 1.08)	**0.022^b^**
**Baseline catheterization data, *median (IQR)***				
Mean right atrium pressure (mmHg)	9 (6, 11)	9 (6.5, 9.5)	8.5 (5.7, 11.2)	0.662^b^
Systolic pulmonary artery pressure (mmHg)	113 (90, 132.5)	97 (87.7, 132)	120 (89, 134)	0.534^b^
Mean pulmonary artery pressure (mmHg)	83 (61.5, 82.5)	68 (52.2, 86.2)	87 (64, 100)	0.138^b^
Pulmonary artery-to-aorta gradient (mmHg)	1.23 (1.1, 1.39)	1.17 (1.09, 1.54)	1.26 (1.17, 1.39)	0.945^b^
Index PVR (Wood units/m^2^)	21 (13.57, 26.23)	17.6 (11.9, 26.2)	22.4 (19.2, 30.32)	0.352^b^
Cardiac Index (L/min/m^2^)	3.2 (2.5, 4.4)	3.02 (2.4, 3.9)	3.65 (2.3, 5.2)	0.537^b^
DAO to LPA distance (mm)*, *median (IQR)*	–	2.1 (0, 3.5)	–	–
**Type of implanted stents, *N* (%)**				
LIFESTREAM (Bard)	6 (46.2)	5 (83.3)	1 (14.3)	**0.029^a^**
Other stents	7 (53.8)	1 (16.7)	6 (85.7)	
VALEO Lifestent (Bard)	4	–	4	
Atrium V12 (Maquet)	1	–	1	
BeGRAFT (Bentley)	1	1	–	
Palmaz Genesis (Cordis)	1	–	1	
Stent nominal diameter (mm), *median (IQR)*	7 (7, 8.5)	7 (7, 7.75)	8 (7, 9)	0.534^b^
Stent length (mm), *median (IQR)*	26 (19.5, 26)	26 (21.2, 36.5)	26 (18, 26)	0.445^b^
High-pressure stent dilation *N* (%)	7 (53.8)	4 (66.7)	3 (42.9)	0.592^a^
Final shunt diameter* (mm), *median (IQR)*	8 (6.7, 8.7)	8.53 (6.9, 8.9)	8 (6.5, 8.1)	0.295^b^
DAO diameter* (mm), *median (IQR)*	12.2 (10, 12.9)	12.2 (11.6, 13.4)	10 (9.8, 12.9)	0.366^b^
Shunt-to-DAO diameter ratio, *median (IQR)*	0.65 (0.57, 0.77)	0.64 (0.57, 0.74)	0.65 (0.57, 0.83)	0.836^b^
Angle between stent and DAO (degrees)*, *median (IQR)*	104 (90, 120)	104 (90, 115)	106 (85, 121)	1^b^
Persistent waist in mid-stent, *N* (%)	6 (46.2)	3 (50)	3 (42.9)	1^a^
Post-procedural PA-to-aorta gradient (mmHg), *median (IQR)*	2 (0, 10)	1 (0, 12.5)	2 (2, 10)	0.534^b^
Intensive care unit stay (days), *median (IQR)*	0 (0, 3)	2.5 (0, 4.25)	0 (0, 0)	0.101^b^

Group 1, non-ductal transcatheter Potts shunt; Group 2: ductal transcatheter Potts shunt. BSA, body surface area; DAO, Descending Aorta; LPA, left pulmonary artery; PA, pulmonary artery; PVR, pulmonary vascular resistance. *Measured in right anterior oblique RAO projection. ^a^Fisher Exact test; ^b^Mann-Whitney *U*-test. Bold values are significant *p*-values.

### Procedure

At the time of the procedure, all children were outpatients. Seven children had ductal TPS (median age, 8.1 years) and 6 children had non-ductal TPS (median age, 9.8 years). The baseline procedural data are shown in Table 1. All implanted stents were pre-mounted on single delivery balloons. LIFESTREAM^®^ stent (Bard Peripheral Vascular, Inc., Tempe, AZ, USA) was implanted in 46.2% of patients and was more frequently used in ductal TPS procedures. Of all implanted stents, 92.3% were made of stainless steel, 92.3% had an open-cell design, and 61.5% were PTFE-covered stents. The median nominal diameter of stents was 7 mm (IQR, 7–8.5). Implanted stents were post-dilated using high-pressure balloons in 7 (53.8%) patients to eliminate residual pressure gradient and achieve the target diameter. Post-dilation balloons were larger than stents’ delivery balloons by a median of 2 mm (IQR, 1–3.5). The median TPS final angiographic diameter was 8 mm (IQR, 6.7–8.7) and the median value of the shunt-to-DAO diameter ratio was 0.65 (IQR, 0.57–0.77). Three stents were protruding in the aorta without a significant gradient across the aortic isthmus. A slight waist in mid-stent was persistent in 6 (46.2%) patients at the end of the intervention. Five patients were transferred to the ICU for postoperative management. Median ICU stay of children with TPS creation was 2.5 days (IQR, 0–4.2 days).

### Immediate and short-term follow-up

All children were uneventfully discharged home with significant improvement in the overall clinical condition that was classified as good in all patients. At discharge, 92.3% of children were considered WHO-FC I or II. The median upper limb/lower limb rest saturation gradient at the time of discharge was 9% (IQR, 6.5–17%). Of the seven patients receiving prostanoid therapy before the procedure, 2 were weaned by hospital discharge, 1 at 3 months, and 1 at 6 months post-procedure.

### Long-term follow-up

**FIGURE 1:**
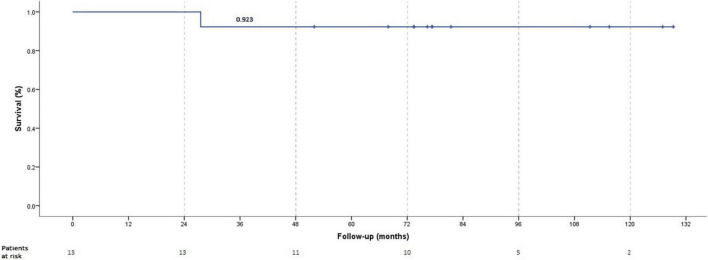
One to 6 years survival rates.

The median follow-up was 77.4 months (IQR, 70.7–113.4). Detailed comparison of clinical, echocardiographic, biological, and pharmacological parameters across follow-up is outlined in Table 2. One 4-year-old patient with ductal TPS died 28.5 months post-procedure from a severe respiratory syncytial virus infection. The overall survival rate was 92.3% at 6 years (Figure 1). The left ventricular function and cardiac output were initially improved and preserved during follow-up. All children caught up to normal growth curves. There was no recurrence of syncope in the 4 patients who experienced syncope before TPS. Two out of nine patients who did not have syncopal episodes in the past, experienced syncope during follow-up.

**TABLE 2:** Detailed comparison of clinical, echocardiographic, biological, and pharmacological parameters across follow-up.

	Baseline, *n* = 13	Discharge, *n* = 13	6 months follow-up, *n* = 12	12 months follow-up, *n* = 13	Latest follow-up, *n* = 13	*P-value*	*P-value* ^‡^
Weight (Kg), *median (IQR)*	26.8 (18, 34.7)	26.8 (18, 34.7)	30.5 (22.4, 36.8)	35.1 (24.8, 40.9)	44 (35.9, 53.4)	**0.022^a^**	**0.005^a^**
I-II, *N* (%)	4 (30.8)	12 (92.3)	10 (83.3)	13 (100)	12 (92.3)	**<0.001^b^**	**0.004^b^**
III-IV, *N* (%)	9 (69.2)	1 (7.7)	2 (16.7)	–	1 (7.7)		
Syncope, *N* (%)	4 (30.8)	–	2 (16.7)	–	2 (15.4)	0.363^b^	0.645^b^
UL oxygen saturation (%), *median (IQR)*	97 (94, 98.5)	98 (96, 99.5)	96 (94.2, 99)	97 (95, 98)	96 (93.5, 98.5)	0.757^a^	0.614^a^
UL/LL oxygen saturation gradient, *median (IQR)*	N/A	9 (6.5, 17)	9.5 (2.7, 18.2)	4 (1, 14.5)	11 (4.5, 15)	0.25^a^	–
TAPSE (mm), *median (IQR)*	18 (12.1, 23)	19 (17, 23.5)	22 (19.3, 23.7)	19.5 (16.5, 21.6)	20.5 (18, 25.7)	0.225^a^	0.06^a^
TAPSE z-score, *median (IQR)*	–0.77 (–3.59, 1.87)	–0.77 (–1.53, 1.59)	0.28 (–1.16, 2.59)	–0.91 (–1.69, 0.98)	–0.47 (–2.92, 0.7)	0.506^a^	0.979^a^
Maximum velocity on stent (m/s), *median (IQR)*	N/A	1.5 (1.5, 2)	2 (1.5, 2.2)	2 (1.6, 2.8)	2 (1.6, 2.3)	0.358^a^	–
NT-pro BNP level (pg/ml), *median (IQR)*	170 (141, 609)	I/D	130 (57.7, 255.2)	59.5 (20.5, 205)	145.5 (27.2, 259.7)	0.112^a^	0.118^a^
6MWT* (m), *median (IQR)*	448 (328.5, 495.3)	451 (355.3, 487)	425 (398, 637)	449 (354, 540)	407 (319, 460)	0.701^a^	0.468^a^
6MWT z-score, *median (IQR)*	–3.69 (–4.65, –2.26)	–3.4 (–4.68, –2.55)	–2.47 (–4.25, –0.13)	–3.73(–4.8, –2.06)	–4.63(–5.71, –4.29)	0.121^a^	0.072^a^
Number of pulmonary hypertension therapies, *median (IQR)*	3 (1.5, 3)	2 (1.5, 3)	2 (2, 2)	2 (2, 2)	2 (2, 3)	0.715^a^	0.545^a^
Type-5 phosphodiesterase (PDE5) inhibitor, *N* (%)	12 (92.3)	11 (84.6)	11 (91.7)	12 (92.3)	12 (92.3)	1^b^	1^b^
Sildenafil total dose (mg/day), *median (IQR)*	60 (35, 60)	60 (50, 60)	60 (55, 60)	60 (57.5, 60)	60 (60, 60)		
Dose adjusted to weight (mg/Kg/day), *median (IQR)*	1.98 (1.71, 2.4)	2.03 (1.71, 2.73)	2.31 (1.73, 3.36)	1.83 (1.55, 2.51)	1.5 (1.25, 1.62)		
Tadalafil total dose (mg/day), *median (IQR)*	–	–	30 (20, –)	40 (40, 40)	40 (40, 40)		
Dose adjusted to weight (mg/Kg/day), *median (IQR)*	–	–	0.62 (0.25, –)	0.74 (0.51, –)	0.56 (0.37, 0.61)		
Endothelin receptor antagonist (ERA), *N* (%)	11 (84.6)	10 (76.9)	11 (91.7)	11 (84.6)	12 (92.3)	0.984^b^	1^b^
Total dose (mg/day), *median (IQR)*	125 (80, 160)	126.5 (92, 168)	125 (80, 128)	128 (96, 160)	173.7 (102.1, 192)		
Dose adjusted to weight (mg/Kg/day), *median (IQR)*	3.68 (3.21, 4.35)	4.01 (3.53, 4.56)	3.69 (3.57, 4.49)	3.97 (3.6, 4.2)	3.88 (3.16, 4.28)		
Prostanoid therapy, *N* (%)	7 (53.8)	5 (38.5)	2 (16.7)	2 (15.4)	4 (30.8)	0.109^b^	0.428^b^
Epoprostinol	3 (42.9)	2 (40)	–	–	–		
Treprostinil	4 (57.1)	3 (60)	1 (50)	1 (50)	1 (25)		
Dose (ng/kg/min)	31 (20, 37.5)	23 (14.7, 31)	35^#^	36.3^#^	32^#^		
Selexipag	–	–	1 (50)	1 (50)	3 (75)		
Dose (μg/day)	–	–	400^#^	2400^#^	2800 (2400, 3000)		
Diuretic therapy	–	–	–	2 (15.4)	2 (15.4)		

^a^Kruskal-Wallis test. ^b^Fisher Exact test. LL, Lower limb; TAPSE, Tricuspid annular plane systolic excursion; UL, upper limb; WHO, world health organization- Functional class; 6MWT, 6 min walking test; N/A, not applicable; I/D, insufficient data. ^‡^Comparison of baseline to last follow-up. *Due to age, the 6MWT could not be performed initially and during follow-up in all patients. ^#^Single value. Bold values are significant *p*-values.

**FIGURE 2:**
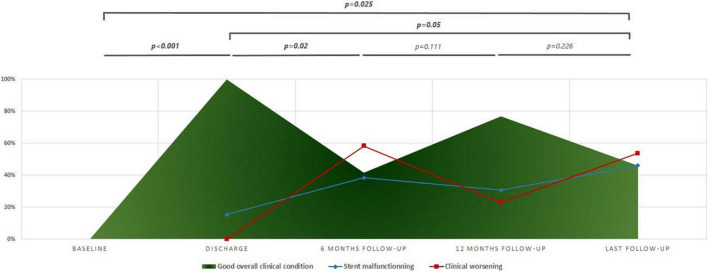
Progression of overall clinical status across follow-up. The clinical worsening is defined by the presence of at least one of the following: (1) worsening in world health organization- Functional class (WHO-FC); (2) NT-pro BNP ≥ 1,400 pg/ml; (3) tricuspid annular plane systolic excursion (TAPSE) ≤ 15 mm; (4) increase or adjunction of diuretics; and (5) adjunction of new pulmonary arterial hypertension (PAH) specific medical therapy. Stent malfunctioning is defined as maximum velocity on stent > 2 m/s.

The remarkable improvement in the baseline overall clinical condition of discharged patients was inconsistent and variable across follow-up (Figure 2). Patients experienced frequent clinical worsening events that were concurrent with stent malfunctioning as detected on ultrasound. Despite this variability in clinical condition, the overall improvement remained significant at the last follow-up when compared to baseline status (*p* < 0.025), and discharge status (*p* = 0.05) (Figure 2). More specifically, improvement in functional status across follow-up was significant (*p* < 0.001). At the last visit, three patients were WHO-FC I, nine patients were WHO-FC II, and one patient was WHO-FC III. When compared with preoperative values, serum NT-proBNP levels diminished from a median of 170 pg/mL (IQR, 141–609) to a median of 145.5 pg/mL (IQR, 27.2–259.7) at last visit but this decrease was not significant (*p* = 0.118). At the last visit, the median 6MWD values and z-scores were not significantly improved when compared with preoperative data. Distribution of TAPSE z-scores across follow-up was also not significant (Table 2). At the last visit, 4/13 (30.8%) patients (2 with non-ductal TPS and 2 with ductal TPS) were receiving triple combination therapy. Weaning of prostanoid therapy weaning was not possible in two patients (one with non-ductal TPS and one with ductal stenting). Prostanoid therapy was stably weaned in 4 patients with non-ductal TPS. It was also weaned in one patient 2 months after non-ductal TPS and then restarted after 52 months for progressive clinical worsening and severe stent malfunctioning. Finally, prostanoid therapy was *de novo* started in another patient 82.8 months after ductal TPS for clinical worsening without stent malfunctioning. Reduction in overall use of PAH-specific medications was not significant.

### Transcatheter reinterventions

**FIGURE 3:**
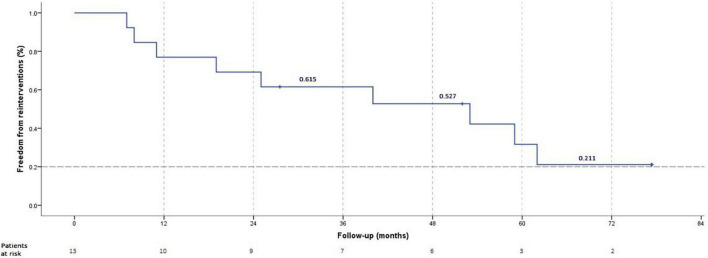
Freedom from re-intervention.

**FIGURE 4:**
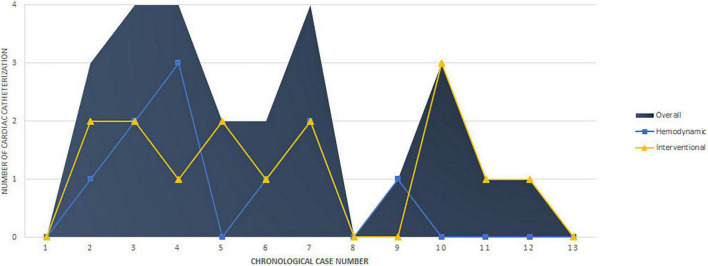
Number and type of re-catheterization according to chronological case number.

The final procedural outcomes are shown in Table 3. The freedom from transcatheter reinterventions was 77% at 1 year and 21% at 6 years (Figure 3). Three patients did not require any redo cardiac catheterization (hemodynamic or interventional) on a follow-up of 28.5, 52, and 77.4 months, respectively. These 3 patients had early ductal TPS (within 2 years of PAH diagnosis) and did not require high-pressure post-dilation of implanted stents during the ductal TPS intervention. Of these three patients, two were in the latter half of the cohort (Figure 4). One patient with non-ductal TPS had one diagnostic hemodynamic redo cardiac catheterization 6 months postoperative for a protruding stent in the aorta and did not require any reintervention. The remaining 9/13 (69.2%) patients had transcatheter reinterventions on implanted stents at a median of 25 months (IQR, 9.5–56) postoperative. These nine patients had a median of 2 (range, 1–3) transcatheter reinterventions per patient. Stent balloon dilatations were performed in 7/13 (53.8%) patients at a median of 25 months (IQR 8–59) postoperative. Endovascular implantations of a second stent were performed in 6/13 (46.1%) patients at a median of 41 months (IQR 24–68) postoperative. We did not identify any stent fracture on follow-up redo catheterizations. Intimal proliferation was noted in two patients. At the latest follow-up, stent malfunctioning was present in 6/13 (46.2%) patients of which 3 had non-ductal TPS. In 2/3 patients with malfunctioning non-ductal TPS, the stent was severely damaged (infolded/distorted geometry) after repetitive transcatheter reinterventions. Interventional therapy was not considered beneficial and both patients are listed for heart-lung transplantation for almost 2 years. Univariate analysis identified that high-pressure post-dilatation of implanted stents during the TPS intervention was alone associated with higher rates of reinterventions (*p* = 0.021). The need for reinterventions was quite similar in both techniques applied for TPS (Table 4).

**TABLE 3:** Final procedural outcomes.

Follow-up (months), *median (IQR)*	77.4 (70.7, 113.4)
Stent malfunctioning at latest follow-up*, *N* (%)	6 (46.2)
Stent protrusion in the aorta (without hemodynamical significance), *N* (%)	3 (23.1)
Stent fracture, *N* (%)	–
Stent intimal proliferation, *N* (%)	2 (15.4)
Re-catheterizations, *N* (%)	10 (76.9)
Number of re-catheterizations per patient, *median (total range)*	2 (0–4)
Control hemodynamic re-catheterizations, *N* (%)	6 (46.1)
Transcatheter re-interventions, *N* (%)	9 (69.2)
Time to first re-intervention (months), *median (IQR)*	25 (9.5, 56)
Number of re-intervention per patient, *median (total range), n* = *9*	2 (1–3)
Stent balloon dilatation, *N* (%)	7 (53.8)
Time to balloon dilatation (months), *median (IQR)*	25 (8, 59)
Re-stenting, *N* (%)	6 (46.2)
Time to re-stenting (months), *median (IQR)*	41 (24, 68)
Listed for heart-lung transplantation, *N* (%)	2 (15.4)
Late death, *N* (%)	1 (7.7)

*Defined as maximum velocity on stent > 2 m/s.

**TABLE 4:** Distribution of study parameters according to the need for reinterventions.

	Re-intervention		*P-value*
		
	No	Yes	
Male gender	2 (50)	5 (55.6)	1^a^
**Demographics at intervention**			
Delay from diagnosis (years)	1.45 (0.65, 3.79)	4.99 (2.28, 7.47)	0.076^b^
Age (years)	5.46 (2, 12.91)	8.92 (8.37, 12)	0.33^b^
Weight (Kg)	23.5 (10.5, 36.2)	26.8 (23.3, 34.5)	0.71^b^
Height (cm)	109 (80, 138)	134 (125, 142)	0.26^b^
**Intervention type**			
Non-ductal transcatheter Potts shunt	1 (25)	5 (55.6)	0.559^a^
Ductal transcatheter Potts shunt	3 (75)	4 (44.4)	
Stent nominal diameter	8 (6.5, 9.5)	7 (7, 8)	0.503^b^
Stent length	23 (18, 35)	26 (22, 26)	0.825^b^
**Type of implanted stents**			
Lifestream	2 (50)	4 (44.4)	1^a^
Other stents	2 (50)	5 (55.6)	
High-pressure balloon post-dilatation	–	7 (77.8)	**0.021^a^**
Persistent waist in mid-stent, *N* (%)	2 (50)	4 (44.4)	1^a^
Final shunt diameter (mm), *median (IQR)*	8 (6.3, 8.6)	8 (6.7, 9)	0.825^b^
Shunt-to-DAO diameter ration, *median (IQR)*	0.83 (0.64, 0.83)	0.59 (0.55, 0.67)	0.076^b^
Angle between stent and DAO (degrees), *median (IQR)*	120 (98, –)	92 (90, 117)	0.373^b^
Post-procedural PA-to-aorta gradient (mmHg), *median (IQR)*	6 (0.5, 10)	2 (0, 10)	0.825^b^
Discharge maximum velocity on stent (m/s), *median (IQR)*	1.75 (1.25, 2.6)	1.5 (1.5, 2)	0.94^b^
Discharge UL/LL oxygen saturation gradient (mmHg), *median (IQR)*	16 (9, 23)	9 (5, 13)	0.26^b^

DAO, Descending Aorta; PA, pulmonary artery. ^a^Fisher Exact test; ^b^Mann-Whitney *U*-test. Bold values are significant *p*-values.

## Discussion

Despite technical challenges, the spatial proximity between the DAO and LPA made the creation of the Potts shunt possible using transcatheter techniques (7, 16–20). This approach was easily performed in the presence of a tiny arterial duct (5, 6). However, the limitations related to the design, delivery profile, size portfolio, and endurance of available stents are now identified as impediments to the long-term durability of early positive procedural outcomes. The choice of initial stent diameter was based on an aggregate consideration of the patient’s age and weight, surgical target diameter, and narrowest arterial duct diameter limiting the straightforward implantation of an adequately sized stent (5, 6, 21). Therefore, we had to gradually enlarge implanted stents by sequential and repetitive balloon inflations to match patients’ growth and disease progression. This technique was somehow helpful in maintaining pulmonary blood flow and limiting lower limb desaturation. However, it appears to be associated with higher rates of reinterventions when theoretically compared to the surgical Potts (4, 5, 10, 11).

The non-ductal TPS required the actual creation of a bridged connection between two distant vessels. The shape of the tightest contact area and the distance between the LPA and DAO were both variable. The absence of dedicated material for this application made the procedure technically challenging and risky (7, 9, 16, 17). The implanted covered vascular stents were not available in all combinations of diameters and lengths for a tailored procedure. Using a covered stent of appropriate diameter resulted in having a stent protruding too much into the aorta, as we experienced in three patients. Sequential stent re-dilation using appropriately sized high-pressure angioplasty balloons was also necessary in other seven patients to first achieve the desired diameter and eliminate the mid-stent waist within the relatively rigid vessel arterial walls, and secondly to abolish the residual pressure gradient (19, 22).

The survival rate in this cohort was better than the one reported in the international Potts registry for the patients discharged home and this can be simply explained by the different design of the two studies and the inclusion criteria of patients (12). The analysis of registry data showed that non-ductal TPS was a risk factor for early mortality but did not identify transcatheter shunts as a significant risk factor for late death or lung transplantation (12). Here, we particularly questioned the durability of the implanted stents during ductal and non-ductal TPS. The most important finding was that the outstanding improvement in overall clinical condition at discharge was variable across follow-up and weaning of prostanoid therapy was not possible in all survivors. This is not the case in survivors of surgical Potts shunts where immediate clinical improvement in survivors is long-lasting and allows progressive weaning of prostanoid therapy as reported elsewhere (4, 5). This finding was not quite surprising since stent malfunctioning was expected as frequently reported in other interventions implicating endovascular stent implantations (22, 23). The aforementioned challenging anatomical conditions of stent implantation during TPS procedure are even additional risk factors for higher rates of stent malfunctioning events.

### Debates, learning points, and room for improvements

Debates on prostanoid therapy and future transplantation candidacy in patients with Potts shunt have been discussed elsewhere (10–12, 24, 25). Three issues are worth debating today.

#### Is there a learning curve impact?

Procedure-related complications and long-term morbidity did not appear consistently less in the latter part of our center experience (Figure 4). Indeed, it was not expected because there was no important change in the implantation techniques and the portfolio limitations of the implanted pre-mounted stents were existent all across the observation period.

#### Should we abandon the transcatheter Potts shunt procedure?

Putting the study findings into perspective, we might conclude that TPS is a bad idea because the need for reinterventions did not vary significantly with the applied technique for TPS. However, we cannot ignore that in-hospital mortality has been solely associated with non-ductal TPS when compared to surgical Potts and ductal TPS in the international Potts registry (12). Aortic stent protrusion is particularly seen in non-ductal TPS and presents an additional surgical difficulty during heart-lung transplantation (6, 11). In addition, considering the reduced interventional complexity, and easier post-procedural care of ductal TPS, resuscitation of a tiny or even closed arterial duct is a reasonable good option. Ductal TPS is a safe and effective transcatheter alternative, when applicable, and should be always considered as a life-saving palliation that delays transplantation, especially for young patients with medically refractory PAH. The real question remains whether surgical Potts should be prioritized even over ductal TPS. The better long-lasting outcomes, the no need for reinterventions, and the easier control for transplantation make somehow the surgical Potts a more reasonable option than the TPS procedure in older children, yet this conclusion deserves further investigation (4, 5, 12).

Previous reports on TPS have commented that patients can often become unstable during the induction of anesthesia (9). Anesthetizing children with severe PAH carries an increased risk of cardiac arrest and death (26, 27). This observation could be considered as an argument against the use of TPS given that the need for multiple reinterventions after this procedure will not only subject small children to increased risk of repeated procedural complications but are also at risk of repeated anesthesia-related perioperative complications.

The shunt size was not addressed in the international Potts registry (12). A direct surgical anastomosis has to be differently assessed than the use of a stent or conduit, both with variable lengths and diameters. In 3D reconstructed views of the LPA and DAO, the tightest DAO-LPA contact area has an elliptic shape stretched along the DAO length and the LPA width (28). The largest diameter of the cross-section’s ellipse represents the presumably suitable stent diameter that can be superimposed upon the tightest contact area. To lower supra-systemic PAH to a systemic level, the diameter and length of the Potts shunt should be adjusted according to the DAO diameter that determines the maximal size of the stent that is possible to deploy. Creating a communication of 90% of the DAO diameter can be initially too large, leading to life-threatening hemodynamics. Therefore, we believe that the target diameter of the TPS should be somewhere between 65 and 75% of the DAO diameter. In unstable patients, a 2-step TPS can be an even better option with the initial communication expanded again once the patient has adjusted to the shunt-related pathophysiology.

#### Is there room for improvement and innovation?

Technically speaking, we believe that ductal-TPS is an already standardized procedure, and the improvement of the technique is somehow limited to the availability of new stent technologies that might implement better material performance, larger functional expansion ranges, flow-reducers, and unidirectional valves as previously evoked for surgical shunts (11, 29). On the other side, the success of the non-ductal TPS is conditioned by both the stability and control of the connection between the fixed DAO and the respiratory-gated movements of the LPA whether during perforation and subsequently throughout the stenting process. The stent bridging technique to create the shunt by reducing the variable space between the 2 vessels turned out somehow inadequate and has been recognized as a bottleneck of the procedure leading to mechanical stent-related complications (e.g., central stent compression, disoriented spatial stent orientation across the vessels, and stent dislodgement) (7). These serious concerns related to the bridged connection have been raised as well for the surgical tube graft and the valved conduit (30). These findings pushed researchers to investigate in experimental models the creation of a connection using magnetic catheters to maintain the required continuous connection and the implantation of a window-like connection device system, spool-shaped self-expanding covered lumen-apposing stents, and most recently the flow regulator devices (18–20). These experimental techniques are promising but none have been tested in humans limiting their actual use. With the leap of faith of researchers and expert interventionists and the continuous advancements in device technology, technical pitfalls can be overcome. Nevertheless, for the present, the resurrection of non-ductal TPS remains conditioned by the availability and efficacy of tailored devices to refine and ease the procedure for widespread acceptance.

### Limitations

This is a report from a single yet expert center. The small number of patients remains the main drawback of this study, yet we present the largest single-center experience with TPS.

## Conclusion

TPS is a pioneering intervention, especially in the absence of an arterial duct. Procedure survivors have satisfactory long-term survival rates but face both clinical and mechanical stent-related long-term morbidities with frequent need for transcatheter reinterventions. High-pressure post-dilatation of implanted stents during TPS intervention for incomplete stent expansion and/or residual pressure gradient appears to be associated with higher rates of reinterventions during follow-up. In the era of continuous advancements in transcatheter technologies, room for innovation is existent and the challenges have to be properly addressed to meet the better long-term procedural outcomes of surgical Potts shunt.

## Data availability statement

The raw data supporting the conclusions of this article will be made available by the authors, upon request, to any qualified researcher.

## Ethics statement

We assert that all procedures contributing to this work comply with the ethical standards of the relevant national guidelines on human experimentation, and with the Helsinki Declaration of 1975, as revised in 2008. Approval from the Institutional Review Board was obtained (MR004: 2021-1004152008). Informed consent was obtained from participants or their legal guardians/next of kin to use and publish their clinical data before their inclusion in the study.

## Author contributions

RH collected all clinical data, performed clinical stratifications, statistical calculations, designed illustrative material, critically analyzed, interpreted the results, and took the lead in writing and revising the entire manuscript. DB, ML, and SM-M supervised the project. All authors discussed the results, read, and approved the final version of the manuscript.

## Abbreviations

DAO: descending aorta
ERA: endothelin receptor antagonists
IQR: interquartile
LPA: left pulmonary artery
NT-proBNP: N-terminal pro-brain natriuretic peptide
PAH: pulmonary arterial hypertension
PDE5I: phosphodiesterase type-5 inhibitors
TAPSE: tricuspid Annular Plane Systolic Excursion
TPS: Transcatheter Potts shunt
WHO-FC: World Health Organization functional class
6MWD: 6-min walk distance.
